# Supplementary material for: Cell-Free DNA Genomic Profiling and Its Clinical Implementation in Advanced Prostate Cancer
Source: Cancers (Basel). 2023 Dec 21;16(1):45. doi: 10.3390/cancers16010045 (PMC10778564; doi:10.3390/cancers16010045)
Supplement: Supplementary file 1 [file cancers-16-00045-s001.zip › Supplementary_Caption.pdf]

Supplementary Tables

Supplementary Table S1: Gene and amplicon list in the Prostate cfDNA AmpliSeq HD Panel.

Supplementary Table S2. Genetic analysis of the PCa cell lines (DU145, VCAP, and LNCaP) by targeted sequencing. Targeted parallel sequencing was performed with Oncomine Comprehensive v3 Panel. The detected allele frequency is given in the brackets.

Supplementary Table S3: Patient cohort and sequencing data.

Supplementary Table S4. Comparison of various commercially available Pan-Tumor cfDNA NGS assays suitable for prostate cancer genomic analysis with our new cfDNA PCa assay.
