# Supplementary material for: Cell-Free DNA Genomic Profiling and Its Clinical Implementation in Advanced Prostate Cancer
Source: Cancers (Basel). 2023 Dec 21;16(1):45. doi: 10.3390/cancers16010045 (PMC10778564; doi:10.3390/cancers16010045)
Supplement: Supplementary file 1 [file cancers-16-00045-s001.zip › Supp_Table S2.pdf]

Supplementary Tables

Supplementary Table S2. Genetic analysis of the PCa cell lines (DU145, VCAP, and LNCaP) by targeted sequencing. Targeted parallel sequencing was performed with Oncomine Comprehensive v3 Panel. The detected allele frequency is given in the brackets.

Cell Line	Mutations
**DU145**	RB1:p.K715*(c.2143A>T)(100%);TP53:p.V274F(c.820G>T) (100%)
**LNCaP**	AR:p.T878A(c.2632A>G) (100%);
**VCaP**	TP53:p.R248W(c.742C>T) (100%); AR gain (27x)
