# Supplementary material for: Cell-Free DNA Genomic Profiling and Its Clinical Implementation in Advanced Prostate Cancer
Source: Cancers (Basel). 2023 Dec 21;16(1):45. doi: 10.3390/cancers16010045 (PMC10778564; doi:10.3390/cancers16010045)
Supplement: Supplementary file 1 [file cancers-16-00045-s001.zip › Supp_Table S4.pdf]

Supplementary Table S4. Comparison of various commercially available Pan-Tumor cfDNA NGS assays suitable for prostate cancer genomic analysis with our new cfDNA assay.

cfDNA Assay	Technology	Approval	Number of Target Genes	Availability
**FoundationOne @ Liquid CDx Assay**	Hybridization-based capture seq	FDA/CE-IVD	324	Centralized
**Guardant 360@ CDx Assay** ***(Guardant Health)***	Hybridization-based capture seq	FDA/CE-IVD	129	Centralized
**TriuSight™ Oncology 500 ctDNA** ***(Illumina)***	Hybridization-based capture seq	RUO	523	In-house
**Oncomine™ Pan-Cancer cfDNA Assay** ***(ThermoFisher)***	Amplicon-based seq	RUO	44 (SNV only)	In-house
**Oncomine™ Precision AssayGx** ***(ThermoFisher)***	Amplicon-based seq	RUO	45 (SNV only)	In-house
**Oncomine Dx Express Test™** ***(ThermoFisher)***	Amplicon-based seq	CE-IVD	42 (SNV only)	In-house
**Prostate Cancer-specific cfDNA Assay** ***(this study)***	Amplicon-based seq	RUO	46 (SNV+AR CNV)	In-house **(this study*)**
